# Supplementary material for: Fibrinogen Alpha Chain as a Potential Serum Biomarker for Predicting Response to Cisplatin and Gemcitabine Doublet Chemotherapy in Lung Adenocarcinoma: Integrative Transcriptome and Proteome Analyses
Source: Int J Mol Sci. 2025 Jan 24;26(3):1010. doi: 10.3390/ijms26031010 (PMC11817752; doi:10.3390/ijms26031010)
Supplement: Supplementary file 1 [file ijms-26-01010-s001.zip › ijms-3390262-supplementary/Figure S1.pdf]

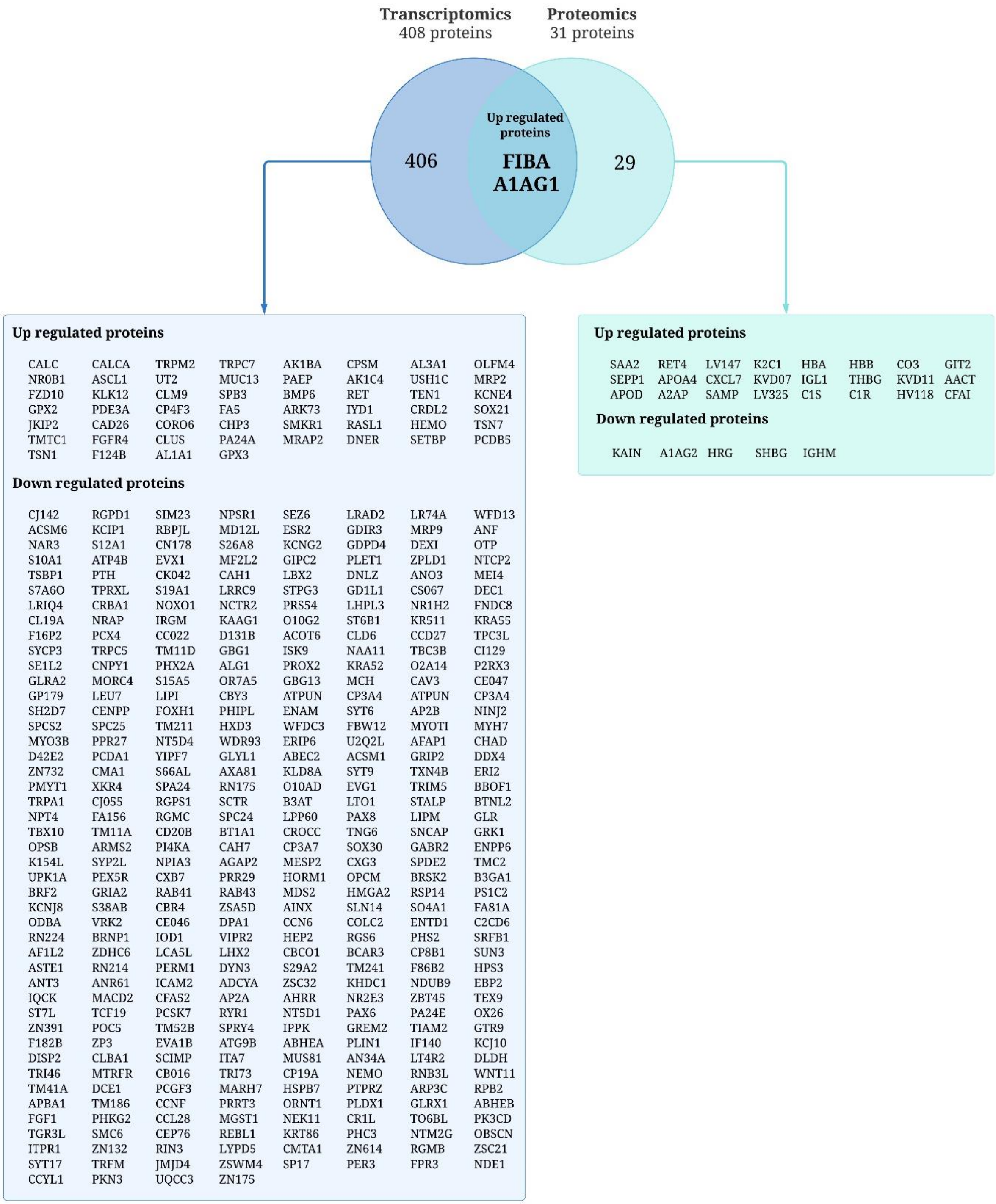

Figure S1. Venn diagram of the profiling in the transcriptomic and proteomic analyses and lists of identified protein. Green and blue circles represent the transcriptome and proteome, respectively. The number of proteins that are overlapping between the two analyses is shown.
