# Supplementary material for: Fibrinogen Alpha Chain as a Potential Serum Biomarker for Predicting Response to Cisplatin and Gemcitabine Doublet Chemotherapy in Lung Adenocarcinoma: Integrative Transcriptome and Proteome Analyses
Source: Int J Mol Sci. 2025 Jan 24;26(3):1010. doi: 10.3390/ijms26031010 (PMC11817752; doi:10.3390/ijms26031010)
Supplement: Supplementary file 1 [file ijms-26-01010-s001.zip › ijms-3390262-supplementary/Table S1.pdf]

Table S1. Clinical characteristic of tissue samples.

Sample Code	Age (year)	Sex	RNA quality		Response
			Yield (ng)	RIN	
**Responder**					
R01	60	F	108	-	PR
R16	40	F	7,740	4.3	PR
R21	48	M	**7,136**	**6.0**	PR
R32	69	F	-	-	PR
R77	62	F	1,318	1.0	PR
R92	57	M	720	7.3	PR
R93	64	F	289	4.1	CR
R119	57	M	431	4.1	PR
R129	72	F	226	-	PR
R190	52	F	412	6.1	PR
**Non-responder**					
N03	65	M	576	7.1	SD
N10	70	F	168	-	PD
N17	66	M	128	-	PD
N18	53	M	615	2.7	SD
N20	39	F	702	-	PD
N25	84	M	-	-	PD
N27	59	M	258	-	PD
N35	41	M	848	-	SD
N47	83	M	136	-	SD
N49	45	F	88	-	PD
N58	62	F	224	-	PD
N82	76	F	**1,376**	**7.4**	PD
N86	76	M	**3,036**	**6.7**	PD
N91	59	F	**3,516**	**5.6**	PD
N106	56	M	93	-	PD
N145	59	M	222	-	SD
N189	61	M	1,104	4.2	SD

Abbreviations: M, male; F, female; RIN, integrity number; CR, complete response; PR, partial response; SD, stable disease; PD, progressive disease
