# Supplementary material for: Fibrinogen Alpha Chain as a Potential Serum Biomarker for Predicting Response to Cisplatin and Gemcitabine Doublet Chemotherapy in Lung Adenocarcinoma: Integrative Transcriptome and Proteome Analyses
Source: Int J Mol Sci. 2025 Jan 24;26(3):1010. doi: 10.3390/ijms26031010 (PMC11817752; doi:10.3390/ijms26031010)
Supplement: Supplementary file 1 [file ijms-26-01010-s001.zip › ijms-3390262-supplementary/Table S2.pdf]

Table S2. Clinical characteristic of tissue-based transcriptome data from the TCGA database.

TCGA database	Age (years)	Sex	Response
**Responder**			
TCGA-55-7283	76	F	PR
TCGA-55-8505	62	M	CR
TCGA-62-A46Y	70	F	CR
TCGA-64-5779	61	M	CR
TCGA-73-4675	59	M	CR
TCGA-86-A4P8	59	F	CR
TCGA-97-8176	63	M	PR
TCGA-MP-A4TD	71	M	CR
**Non-responder**			
TCGA-53-7624	40	F	PD
TCGA-55-7227	77	M	SD
TCGA-55-A48Z	60	F	SD
TCGA-69-7974	54	F	PD
TCGA-95-A4VK	74	F	SD
TCGA-NJ-A7XG	49	M	SD

Abbreviations: M, male; F, female; CR, complete response; PR, partial response; SD, stable disease; PD, progressive disease.
